# Whole genome sequence analysis of *Aeromonas* spp. isolated from ready-to-eat seafood: antimicrobial resistance and virulence factors

**DOI:** 10.3389/fmicb.2023.1175304

**Published:** 2023-06-30

**Authors:** Hye-Jeong Lee, Julia E. Storesund, Bjørn-Tore Lunestad, Sunniva Hoel, Jørgen Lerfall, Anita Nordeng Jakobsen

**Affiliations:** ^1^Department of Biotechnology and Food Science, Norwegian University of Science and Technology, Trondheim, Norway; ^2^Section for Contaminants and Biohazards, Institute of Marine Research, Bergen, Norway

**Keywords:** *Aeromonas*, antimicrobial resistance, virulence factors, mobile genetic elements, whole genome sequences, multilocus phylogenetic analysis

## Abstract

*Aeromonas* are widespread in aquatic environments and are considered emerging pathogens in humans and animals. Multidrug resistant (MDR) *Aeromonas* circulating in the aquatic environment and food production chain can potentially disseminate antimicrobial resistance (AMR) to humans *via* the foodborne route. In this study, we aimed to investigate AMR and virulence factors of 22 *Aeromonas* strains isolated from ready-to-eat (RTE) seafood. A multilocus phylogenetic analysis (MLPA) using the concatenated sequences of six housekeeping genes (*gyrB*, *rpoD*, *gyrA*, *recA*, *dnaJ*, and *dnaX*) in the 22 *Aeromonas* genomes and average nucleotide identity (ANI) analysis revealed eight different species; *A. caviae*, *A. dhakensis*, *A. hydrophila*, *A. media*, *A. rivipollensis*, *A. salmonicida*, *A. bestiarum*, and *A. piscicola*. The presence of virulence genes, AMR genes and mobile genetic elements (MGEs) in the *Aeromonas* genomes was predicted using different databases. Our data showed that the genes responsible for adherence and motility (Msh type IV pili, tap type IV pili, polar flagella), type II secretion system (T2SS) and hemolysins were present in all strains, while the genes encoding enterotoxins and type VI secretion system (T6SS) including major effectors were highly prevalent. Multiple AMR genes encoding *β-*lactamases such as *cphA* and *bla_OXA_* were detected, and the distribution of those genes was species-specific. In addition, the quinolone resistance gene, *qnrS2* was found in a IncQ type plasmid of the *A. rivopollensis* strain A539. Furthermore, we observed the co-localization of a class I integron (*intl1*) with two AMR genes (*sul1* and *aadA1*), and a Tn521 transposon carrying a mercury operon in *A. caviae* strain SU4-2. Various MGEs including other transposons and insertion sequence (IS) elements were identified without strongly associating with detected AMR genes or virulence genes. In conclusion, *Aeromonas* strains in RTE seafood were potentially pathogenic, carrying several virulence-related genes. *Aeromonas* carrying multiple AMR genes and MGEs could potentially be involved in the dissemination and spread of AMR genes to other bacterial species residing in the same environment and possibly to humans. Considering a One-Health approach, we highlight the significance of monitoring AMR caused by *Aeromonas* circulating in the food chain.

## 1. Introduction

*Aeromonas* are Gram-negative bacteria, ubiquitous in aquatic environments, including estuarine and brackish water (Martin-Carnahan and Joseph, 2005). Psychrophilic *A. salmonicida* and some mesophilic *Aeromonas* are responsible for fish diseases such as furunculosis and motile *Aeromonas* septicemia (MAS), while many species are opportunistic human pathogens (Janda and Abbott, 2010; Beaz-Hidalgo and Figueras, 2013). The genus *Aeromonas* comprises at least 31 species, and among them, *A. hydrophila*, *A. caviae*, and *A. veronii* are the major species that frequently involved in human gastroenteritis and extraintestinal infections (Fernández-Bravo and Figueras, 2020). Although the role of *Aeromonas* as a true enteropathogen has been controversial, several studies have suggested that more attention should be given to the genus *Aeromonas* as emerging foodborne pathogens (Teunis and Figueras, 2016; Wu et al., 2018; Hoel et al., 2019). *Aeromonas* have occasionally been recognized as the source of foodborne outbreaks (Zhang et al., 2012; Ventura et al., 2015). The occurrence of mesophilic *Aeromonas* in water and food including ready-to-eat seafood (RTE) has frequently been reported, and their pathogenic potential has been determined based on the analysis of virulence-associated toxin genes (Pablos et al., 2011; Hoel et al., 2017; Lee et al., 2021). The pathogenesis of *Aeromonas* is complex and multifactorial as several virulence factors related to adherence, motility, secretion, and toxins are involved (Tomás, 2012; Fernández-Bravo and Figueras, 2020).

Antimicrobial resistance (AMR) is an emerging threat to public health around the globe. The extensive use and abuse of antimicrobial drugs for humans and animals, and the spread of resistant bacteria within and between these sectors and the environment, has contributed to the increased emergence and spread of AMR (Mcewen and Collignon, 2018). Resistant bacteria residing in the food chain can spread to humans *via* the foodborne route (Founou et al., 2016; EFSA Panel on Biological Hazards (BIOHAZ), 2021). In the food chain, food animals including fish, vegetables, as well as food-producing environments are considered important reservoirs of resistant bacteria (EFSA Panel on Biological Hazards (BIOHAZ), 2021). Bacteria can be intrinsically resistant or acquire new resistance mechanisms by obtaining genetic materials located in mobile genetic elements (MGEs) such as plasmids or transposons, where the latter phenomenon is known as horizontal gene transfer (HGT; Piotrowska and Popowska, 2015; Founou et al., 2016).

The usage of antimicrobial drugs for humans and animals in Norway is very restrictive compared to most other countries (NORM/NORM-VET, 2020). Only two antimicrobial agents are legally used in aquaculture in Norway, and a substantial decrease in antimicrobial usage has been implemented in aquaculture since the top in 1987 (Love et al., 2020). However, antibiotic residues can reach aquatic environments, not only through use in aquaculture, but also through routes like agricultural run-off or improper wastewater treatment (Sanseverino et al., 2018). The occurrence of multidrug resistant (MDR) *Aeromonas* strains has been reported from RTE seafood on the Norwegian market (Lee et al., 2021) as well as other types of food including marine bivalves (Stratev and Odeyemi, 2016; Albini et al., 2022). In addition, *Aeromonas* have been shown to carry multiple AMR genes as well as MGEs, implying their potential to transfer AMR genes to other bacterial species (Piotrowska and Popowska, 2015; Dubey et al., 2022a,b).

Nevertheless, available information on AMR of *Aeromonas* is limited since it is based on phenotypic resistance patterns, or the screening of target AMR genes. Thus, the characterization of AMR genes and MGEs of MDR *Aeromonas* would be necessary to understand their resistance mechanisms and assess their potential to spread AMR genes. With the development of whole genome sequencing (WGS) technology and bioinformatics tools, it is possible to perform a more comprehensive analysis on obtaining AMR and virulence gene profiles in bacterial genomes. In recent years, comparative genomic analysis on the AMR or virulence genes has enabled us to evaluate the potential role of *Aeromonas* in spreading AMR to other bacteria, as well as to predict the pathogenic potential of the *Aeromonas* (Dubey et al., 2022a,b; Erickson et al., 2023; Song et al., 2023). However, to our knowledge, such analysis has not been conducted on the *Aeromonas* residing in the food chain, particularly in relation to RTE seafood. Therefore, in this study, whole genome sequences of 22 *Aeromonas* isolated from RTE seafood were obtained to explore the presence of all AMR and virulence genes in their genomes. In addition, the presence of MGEs was examined to investigate their potential to disseminate AMR or virulence genes to other bacterial species.

## 2. Materials and methods

### 2.1. Bacterial collection

In total, 79 *Aeromonas* isolates were investigated in this study. Among the 79 isolates, 26 were previously isolated from retail sushi products (Hoel et al., 2015), and 43 were isolated from different types of RTE seafood including retail sushi, salmon loins, oysters, and scallops (Lee et al., 2021). All of these 69 isolates were identified as *Aeromonas* spp. based on partial *gyrB* gene sequencing in previous studies (Hoel et al., 2017; Lee et al., 2021). In addition, ten presumptive *Aeromonas* isolates originating from a salmon processing environment (SPE) were donated by Thomassen et al. (2022, 2023), and subjected to *gyrB* gene sequencing for species identification in the present study.

### 2.2. Species identification by partial *gyrB* gene sequencing

Genomic DNA was extracted from 1 mL of overnight cultures grown in Tryptone Soy Broth (TSB; Oxoid, Oslo, Norway) at 37°C using the protocol for Gram-negative bacteria in the GeneJET Genomic DNA Purification Kit (Thermo Fisher Scientific, Oslo, Norway). For PCR amplification, the primers gyrB3F (5′-TCCGGCGGTCTGCACGGCGT-3′) and gyrB14R (5′-TTGTCCGGGTTGTACTCGTC-3′) were used to amplify an approximately 1,100 bp *gyrB* gene (Yáñez et al., 2003). All PCR reactions were performed with 25 μL containing 1 x PCR buffer (1.5 mM MgCI_2_), 200 μM of each nucleotide, 0.4 μM each primer, 2.5 U Taq polymerase (Qiagen, Oslo, Norway) and 50–100 ng DNA template. PCR amplification was as follows: initial denaturation at 95°C for 15 min, 35 cycles of denaturation at 95°C for 30 s, annealing at 52°C for 30 s, and extension at 72°C for 60 s, followed by a final extension at 72°C for 7 min. PCR products were visualized by electrophoresis in a 1.5% agarose gel (SeaKem, Lonza Group Ltd., Basel, Switzerland) in 1 × TAE buffer. PCR products were purified with the GeneJET PCR Purification Kit (Thermo Fisher Scientific). DNA sequencing was performed by Eurofins Genomics (Ebersberg, Germany), and the sequences were compared with available sequences (> 97% nucleotide BLAST similarity) in the GenBank database in the National Center for Biotechnology Information (NCBI). A phylogenetic analysis was conducted based on the *gyrB* gene sequence of the 79 isolates and 11 relevant reference strains by the neighbor-joining (NJ) method with bootstrapping (1,000 replicates) using MEGA 11 version 11.0.10 (Tamura et al., 2021), according to the method described by Lee et al. (2021). The list of the reference strains is shown in Supplementary Table S1A.

### 2.3. Antimicrobial susceptibility testing (AST)

Among the 79 isolates, AST was performed for 36 isolates from Hoel et al. (2015) and Thomassen et al. (2022, 2023), while the antimicrobial susceptibility profile of 43 *Aeromonas* isolates was obtained from the previous study by Lee et al. (2021). AST was conducted by a disk diffusion method according to the recommendation of the Clinical and Laboratory Standards Institute (CLSI, 2016). In brief, the fresh inoculum of each isolate was evenly spread by a sterile cotton swab on Mueller-Hinton agar (MHA; Oxoid, Oslo, Norway). A maximum of five antibiotic disks were placed on each plate on the surface before incubation at 35°C for 16–18 h. After incubation, the diameter of the inhibition zone around the disks was measured, and the degree of susceptibility was categorized as sensitive, intermediate, or resistant according to CLSI criteria (CLSI, 2014, 2016). The resistance pattern of each isolate was examined against 15 antimicrobials belonging to 9 antimicrobial classes including aminoglycosides: gentamicin (10 μg) and tobramycin (10 μg), amphenicols: florfenicol (30 μg) and trimethoprim/sulfamethoxazole (1.25/23.75 μg), carbapenems: imipenem (10 μg) and meropenem (10 μg), cephalosporins: cefotaxime (30 μg) and ceftriaxone (30 μg), macrolides: erythromycin (15 μg) and oxolinic acid (2 μg), penicillin: ampicillin (10 μg) and mecillinam (10 μg), quinolones: ciprofloxacin (5 μg), tetracyclines: doxycycline (30 μg) and tetracycline (30 μg; Oxoid). Based on the antimicrobial susceptibility pattern, MDR strains were defined as being resistant to at least one antimicrobial agent in three or more antimicrobial classes (Magiorakos et al., 2012).

### 2.4. WGS and genome assembly

To select the isolates of interest for WGS analysis, the 79 *Aeromonas* isolates were first divided into eight different groups representing eight different *Aeromonas* species based on the *gyrB* gene sequences. Depending on their source of isolation and antimicrobial susceptibility pattern, 22 *Aeromonas* isolates were chosen for WGS. Total genomic DNA was extracted from 1 mL of overnight cultures grown in TSB (Oxoid) at 37°C, using the Genomic Micro AX Bacteria Gravity kit (A&A biotechnology, Poland) according to the manufacturer’s protocol. The quality of the DNA was checked on agarose gel, and DNA concentrations were estimated by spectrophotometric measurement using BioTek PowerWave XS (Winooski, VT, United States), Take3 plate and Gen5 2.0 software (BioTek Instruments Inc., Winooski, VT, United States). DNA samples were shipped on ice overnight to the Norwegian Sequencing Center (Ullevål University Hospital, Oslo, Norway) for WGS. Sequencing libraries were prepared using the Nextera DNA Flex Library Prep kit (Illumina, United States). Sequencing was performed using Illumina MiSeq platform (Illumina, United States), with 2 × 300 bp chemistry. The raw paired-end reads were cleaned and quality trimmed using BBDuk^1^ and assembled using SPAdes v3.15.4 (Bankevich et al., 2012). Draft genome assemblies were annotated using the NCBI Prokaryotic Genome Annotation Pipeline (Tatusova et al., 2016). The genome assemblies of the 22 isolates were deposited in the NCBI Genbank with accession numbers (JAOPLB000000000-JAOPLW000000000) (Supplementary Table S1B).

### 2.5. Multilocus phylogenetic analysis (MLPA) and average nucleotide identity (ANI) analysis

The housekeeping gene sequences of 10 reference strains (either type strains or representative strains) were retrieved from NCBI Genbank (Supplementary Table S1A). For MLPA, six housekeeping gene sequences (*gyrB*, *rpoD*, *gyrA*, *recA*, *dnaJ*, and *dnaX*) were extracted from each of the 22 *Aeromonas* genomes by blasting the nucleotide sequences of 10 reference strains against the assembled genomes. Multiple sequence alignments of each housekeeping gene were performed using CLUSTAL W (Thompson et al., 1994) implemented in MEGA11. The aligned sequences were then concatenated and re-aligned to concatenated sequences (4,172 bp). Genetic distances between the sequences were calculated using Kimura’s two-parameter model (Kimura, 1980) and a phylogenetic tree was constructed by the NJ method with bootstrapping (1,000 replicates) using MEGA 11 (Tamura et al., 2021). To confirm the tree topology, a phylogenetic tree was also created using the maximum-likelihood (ML) method with bootstrapping (100 replicates). To verify the taxonomy, ANI values between the genomes including 22 isolates and eight reference strains were calculated using FastANI (Jain et al., 2018). ANI matrix was clustered by scripy’s UPGMA and an ANIclustermap was created using the ANIclustermap pipeline (Shimoyama, 2022). The list of the reference genomes used for ANI analysis is included in Supplementary Table S1C.

### 2.6. Prediction of virulence genes, AMR genes, and MGEs

Genes associated with pathogenic bacteria virulence factors were identified by using VFanalyzer based on the virulence factors database (VFDB; Liu et al., 2019), where the threshold for virulence factor detection was set at 80%. The profile of virulence factors was visualized using the package pheatmap in R studio v.4.2.2 (R Core Team, 2022). The presence of AMR genes in bacterial genomes was predicted using the NCBI AMRFinderPlus v3.10.45 (Feldgarden et al., 2019), and ResFinder v4.1 (Bortolaia et al., 2020) with default settings (the threshold for AMR gene identification was set at 90%). PlasmidFinder v2.1 (Carattoli et al., 2014) was used to identify plasmids in the genome assemblies where the threshold for plasmid identification was set at 80%. MGEs were identified using MobileElementFinder v1.0.3 (Johansson et al., 2021) with default settings (minimum sequence identity of 90%). Circular maps of chromosomes and plasmids were visualized using the Proksee software (Grant et al., 2023).

## 3. Results

### 3.1. MLPA and ANI value

**Figure 1:**
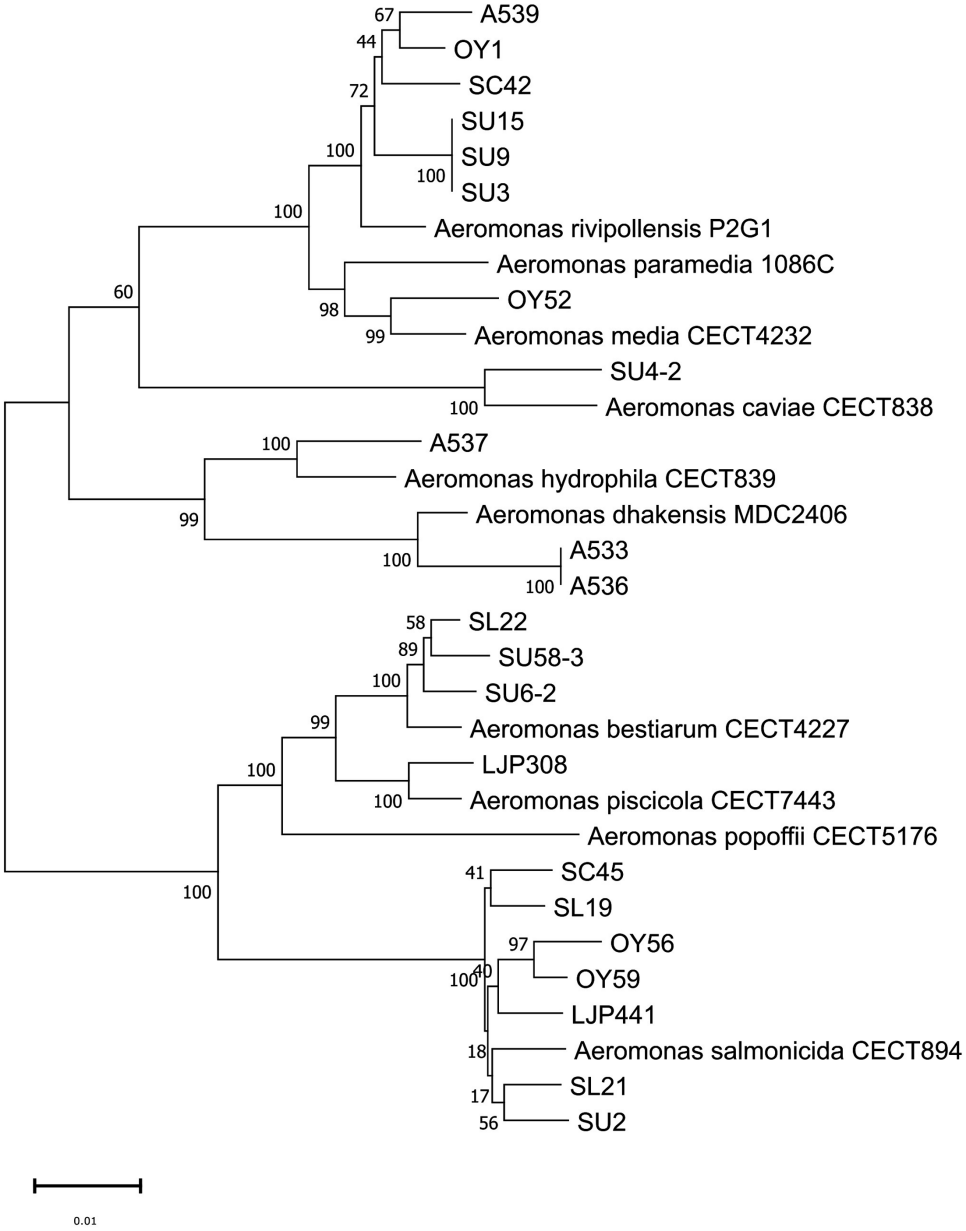
Multilocus phylogenetic analysis (MLPA) based on the concatenated sequence of six housekeeping genes (*gyrB*, *rpoD*, *gyrA*, *recA*, *dnaJ*, and *dnaX*, in total 4,172 bp) by the neighbor-joining method showing the relationship of 22 *Aeromonas* isolates and 10 reference strains. Numbers at nodes indicate bootstrap values (1,000 replicates) and the scale bar, 0.01 indicates the evolutionary distance of nucleotide substitution per site. The sequence data of the reference strains was retrieved from NCBI Genbank (Supplementary Table S1).

The phylogenetic tree based on the *gyrB* gene sequences (a continuous stretch of 929 bp) of the 79 isolates and relevant reference strains was constructed by the NJ method for species identification (Supplementary Figure S1). The MLPA of the 22 *Aeromonas* isolates selected for WGS and relevant reference strains was performed based on the concatenated sequences (4,172 bp) of six housekeeping genes (*gyrB*, *rpoD*, *gyrA*, *recA*, *dnaJ* and *dnaX*) by the NJ method (Figure 1). An identical tree-topology was obtained by the phylogenetic analysis using the ML method, confirming the robustness of the NJ tree (data not shown). The constructed tree comprised two main clusters; one cluster included the species *A. salmonicida*, *A. piscicola*, *A. bestiarum*, and *A. popoffii*, and the other cluster included the species *A. hydrophila*, *A. dhakensis*, *A. caviae*, *A. media*, *A. rivipollensis*, and *A. paramedia* [one of the *A. media* species complex suggested by Talagrand-Reboul et al. (2017)]. All isolates of interest clustered to the respective reference strains. In addition, the same clustering of the isolates to the reference strains, with one exception, was observed in further phylogenetic analysis based on each of the housekeeping gene sequence *gyrB* (908 bp)*, rpoD* (657 bp)*, gyrA* (709 bp)*, recA* (598 bp)*, dnaJ* (800 bp) and *dnaX* (500 bp; data not shown). One exception was the clustering of SU58-3 with *A. piscicola* observed in the tree based on the *dnaX* gene sequence, while each of the other five gene sequences of SU58-3 clustered with *A. bestairum*.

**Figure 2:**
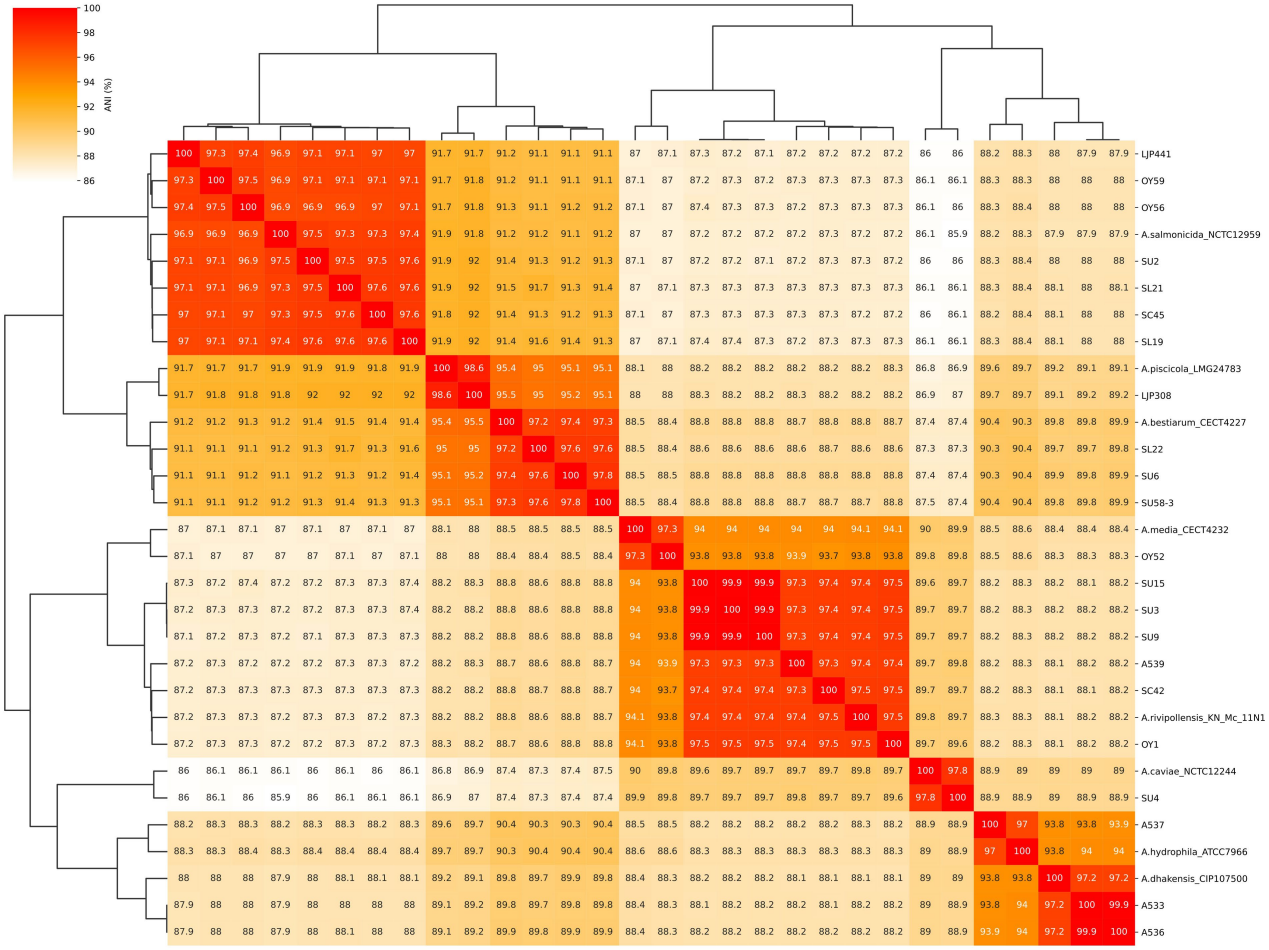
Heatmap of average nucleotide identity (ANI) values among the 22 *Aeromonas* isolates and 8 reference genome assemblies. The numbers represent the ANI values (%) between two genome sequences. The reference strains (NCBI Genbank accession number) used in this study were *Aeromonas bestiarum* CECT 4227 (NZ_CDDA00000000), *A. caviae* NCTC 12244 (NZ_LS483441), *A. dhakensis* CIP 107500 (NZ_CDBH00000000), *A. hydrophila* ATCC 7966 (NC_008570), *A. media* CECT 4232 (NZ_CDBZ00000000), *A. piscicola* LMG 24783 (NZ_CDBL00000000), *A. rivipollensis* KN-Mc-11N1(NZ_UAPT00000000) and *A. salmonicida* NCTC 12959 (NZ_UAPT00000000).

Considering the ANI cutoff value of ≥ 96% for strains belonging to the same species, the ANI values between the 22 isolates and reference genome (Figure 2) supported the phylogenetic clustering observed in the tree shown in Figure 1. Our ANI analysis between 22 isolates and reference genomes confirmed the eight different species among our isolates. In addition, an ANI value of 99.9–100% was observed between the two isolates A533 and A536 from retail sushi product in 2015, as well as among the three isolates SU3, SU9 and SU15 from retail sushi in 2019, indicating that those isolates share high enough genomic similarity to be considered identical strains. Thus, only one genome representing the identical strains (A533 and SU3, respectively) was included in the final dataset of 19 *Aeromonas* strains for the prediction of virulence and AMR genes.

### 3.2. Virulence factors

**Figure 3:**
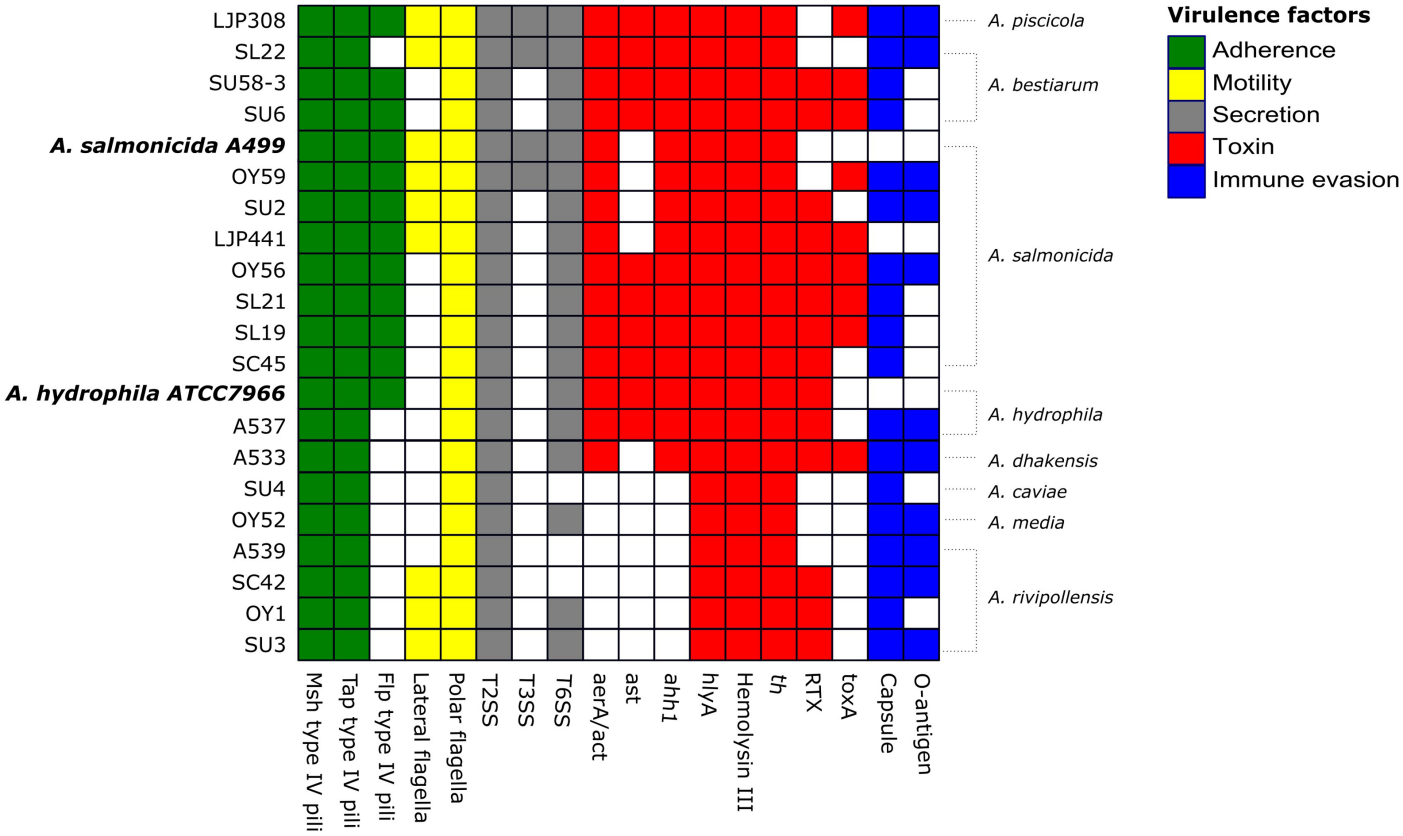
Heatmap showing the distribution of virulence factors across the 19 *Aeromonas* strains and 2 reference strains *A. hydrophila* subsp. *hydrophila* ATCC 7966 (NC_008570) and *A. salmonicida* subsp. *salmonicida* A449 (NC_009348, NC_009350). The presence of each virulence factor belonging to five categories is highlighted in different colors as follows; adherence (in green), motility (yellow), secretion (grey), toxin (red) and immune evasion (blue), and the absence of the virulence factor is marked in white color.

Major virulence factors examined in this study include five categories; (i) adherence: the genes encoding type I and type IV pili, (ii) motility: the genes encoding polar and lateral flagella, (iii) immune evasion: the gene encoding capsules and lipopolysaccharide (LPS) O-antigens, (iv) secretion systems: the genes encoding type II secretion system (T2SS), type III secretion system (T3SS), and type VI secretion system (T6SS), and (v) toxins: the genes encoding cytotoxic and cytotonic enterotoxins, and exotoxins. Over 250 genes encoding multiple virulence factors were identified among the 19 *Aeromonas* strains, and virulence gene profiles of the 19 genomes were compared to the profiles of two reference genomes: *A. hydrophila* subsp. *hydrophila* ATCC 7966, which is a well-characterized type strain originally isolated from a tin of milk with a fishy odor (Seshadri et al., 2006), and *A. salmonicida* subsp. *salmonicida* A449, which is a type strain isolated from a brown trout with furunculosis (Reith et al., 2008) (Figure 3). Overall, the strains belonging to *A. piscicola*, *A. bestiarum*, *A. salmonicida*, *A. hydrophila*, and *A. dhakensis* contained more virulence factors than the strains belonging to *A. caviae*, *A. media*, and *A. rivipollensis*.

#### 3.2.1. Adherence and motility

In this study, the presence of three distinct type IV pili was examined in 19 *Aeromonas* strains and those included were (i) mannose-sensitive haemagglutinin (Msh) pili, (ii) Tap pili, and (iii) Flp pili. Two reference strains contained the genes encoding all three type IV pili. Msh pili was also present in all 19 strains, while only ten contained a complete 17 gene cluster (*msh-A,-B,-C,-D,-E,-F,-G,-H,-I,-I1,-J,-K,-L,-M,-N,-O,-P,-Q*) encoding the Msh pili and those were the strains *A. hydrophila* A537, *A. rivipollensis* OY1, SU3, SC42, A539, *A. salmonicida* SU2 OY56, LJP441, and *A. bestiarum* SU6, SU58-3. The rest of the strains lacked some of the genes such as *mshA* or *mshQ*. In addition, over 14 genes including *tapD* encoding Tap pili, were found in all 19 strains, while Flp type IV pili were only detected in the *A. salmonicida*, *A. piscicola* and *A. bestiarum* strains (except for SL22). Like two references strains, type I pili were detected in 12 strains belonging to *A. dhakenis*, *A. caviae*, *A. piscicola*, *A bestiarum*, and *A. salmonicida* (except for SL21).

Moreover, over 50 genes encoding polar flagella were detected in all 19 strains as well as in two reference strains. Two major flagellin genes required for optimal polar flagella functions, *flaA* and *flaB*, were identified in most of the strains except for four strains *A. rivipollensis* A539, SU3, SC42, and *A. caviae* SU4-2. Other genes encoding flagellar motor components including *fliG*, *fliM*, *fliN*, *motX*, *motY*, *pomA(A_2_),* and *pomB(B_2_)* were found in all 19 strains. On the contrary, about 36 genes encoding lateral flagella including *laf* genes were detected in eight strains including *A. rivipollensis*, SU3, SC42, OY1, *A. salmonicida* SU2, OY59, LJP441, *A. bestiarum* SL22, and *A. piscicola* LJP308. The genes encoding the lateral flagella were observed in the reference strain *A. salmonicida* A449, but not in the *A. hydrophila* ATCC 7966.

#### 3.2.2. Immune evasion

Most of the *Aeromonas* strains contained genes encoding capsules except for *A. salmonicida* LJP441. Genes encoding the LPS O-antigens were identified in 11 strains, including *A. dhakensis* A533, *A. hydrophila* A537, *A. media* OY52, *A. rivipollensis* A539, SU3, SC42, *A. salmonicida* SU2, OY56, OY59, *A. bestiarum* SL22 and *A. piscicola* LJP308. On the contrary, none of the reference strains had the genes encoding the capsules or LPS O-antigens.

#### 3.2.3. Secretion systems

Genes encoding T2SS were detected in all examined strains including two reference strains. Like the *A. hydrophila* ATCC7966, most of the strains did not contain the genes encoding T3SS. Only three strains including *A. bestiarum* SL22, *A. piscicola* LJP308 and *A. salmonicida* OY59 had over 40 genes encoding T3SS, like the *A. salmonicida* A449; however, the bifunctional toxin gene, *aexT*, was only detected in the reference strain. Moreover, over 20 genes encoding T6SS, including major effectors such as *hcp (or hcp1)*, *vgrG1*, *vasH*, or *vasK*, were detected in most of *Aeromonas* strains, except for three strains including *A. rivipollensis* A539, SC42, and *A. caviae* SU4-2. Both reference strains contained the genes encoding T6SS; however, some of the effector genes such as *hcp* and *vgG* were absent in the genome of *A. salmonicida* A499.

#### 3.2.4. Toxin genes

All 19 *Aeromonas* strains contained the genes encoding hemolysin HlyA (*hlyA*), hemolysin III, and thermostable hemolysin, like both reference strains. Genes encoding the extracellular heat-liable hemolysin (*ahh1*) and the aerolysin AerA/cytotoxic enterotoxin Act (*aerA/act*) were also detected in both reference strains and most of our strains, except for the *A. caviae*, *A. media* and *A. rivipollensis*. The heat-stable cytotonic enterotoxin gene (*ast*) was found in the *A. hydrophila* ATCC 7966 as well as nine strains including *A. hydrophila* A537, *A. bestiarum* SU6, SU58-3, SL22*, A. piscicola* LJP308 and *A. salmonicida* SL19, SL21, SC45, OY56. In addition, six genes encoding the repeat toxins (RTX: *rtx-A,-B,-C,-D,-E,-H*) were detected in the *A. hydrophila* ATCC 7966 and ten strains including *A. hydrophila* A537, *A. dhakensis* A533, *A. bestiarum* SU6, SU58-3, and all of the *A. salmonicida* strains except for OY59. Three strains of *A. rivipollensis* SU3, SC42, OY1 possessed only the *rtxA* gene. Furthermore, the exotoxin A (ETA) gene, *toxA* was detected in nine strains including *A. dhakensis* A533, *A. salmonicida* SL19, SL22, OY56, OY59, LJP441, *A. bestiarum* SU6, SU58-3, and *A. piscicola* LJP308, unlike the reference strains.

#### 3.2.5. Other virulence factors

The *adeG* gene encoding efflux pump autoinducer, related to biofilm formation was detected in *A. bestiarum* strain SU58-3. Iron uptake genes, *basG* or *basB* were detected in two strains *A. dhakensis* A533 and *A. hydrophila* A537. The catalase-peroxidase gene, *katG*, associated with stress adaptation was detected in all of the *A. caviae*, *A. media* and *A. rivipollensis* strains. The *neuB2* gene encoding O-linked flagellar glycosylation was detected in two strains *A. media* OY52 and *A. salmonicida* SU2. More detailed information on virulence gene profile is available in Supplementary Table S3.

### 3.3. AMR

Phenotypic antimicrobial susceptibility profiles of 79 *Aeromonas* isolates (Supplementary Table S2) showed that all isolates were resistant to ampicillin as expected. Resistance to erythromycin, florfenicol and oxolinic acid was observed in 57, 48, and 22% of the strains, respectively. Reduced susceptibility to oxolinic acid was observed in 34% of the isolates. In addition, resistance (10%) or reduced susceptibility (11%) to imipenem was mostly observed in *A. salmonicida*, *A. bestiarum* and *A. dhakensis* strains. Resistance to cefotaxime was found in the *A. rivipollensis* strain SU2 and SU3, while reduced susceptibility to cefotaxime and ceftriaxone was observed in about 30% of the strains. One *A. salmonicida* strain (OY59) was resistant to both imipenem and meropenem, and another two *A. salmonicida* strains (OY60 and 61) showed reduced susceptibility to tobramycin. The *A. caviae* strain SU4-2 showed reduced susceptibility to trimethoprim/sulfamethoxazole. None of the *Aeromonas* strains were resistant to mecillinam, ciprofloxacin, doxycycline, tetracycline, or gentamycin. Moreover, about 58% of the *Aeromonas* strains were considered MDR. Most MDR strains were originally isolated from RTE seafood, and they were mainly resistant to ampicillin (penicillins), erythromycin (macrolides), florfenicol (amphenicols) and oxolinic acid (quinolones). On the other hand, none of the strains originated from the SPE was considered MDR.

Furthermore, WGS confirmed that all 19 *Aeromonas* strains contained multiple AMR genes in their genomes and revealed the presence of different classes of *β*-lactamases (Table 1). The class B metallo-*β-*lactamases (MBL) group was dominated by *cphA1* and *cphA5* found in the *A. salmonicida*, *A. bestiarum*, and *A. piscicola* strains, as well as *cphA2* detected in the *A. dhakensis* and *A. hydrophila* strains. However, genes belonging to class B MBL group were not detected in any of the *A. caviae*, *A. media* and *A. rivipollensis*. Genes belonging to class C *β-*lactamases (*bla_AQU_*, *bla_MOX_*, and *cepS*) were detected only in four strains including *A. caviae* SU4-2, *A dhakensis* A533, *A. hydrophila* A537, and *A. media* OY52. All 19 *Aeromonas* strains contained different types of *bla_OXA_* genes belonging to the class D *β-*lactamases group. Identified *bla_OXA_* type genes were species-specific as following; *bla_OXA-12_* (in *A. dhakensis*, *A. hydrophila*), *bla_OXA-780_* (in *A. caviae*), *bla_OXA-427_* (in *A. media* and *A. rivipollensis*), *and bla_OXA-956_* (in *A. salmonicida*, *A. bestiarum*, and *A. piscicola*). The class A *β-*lactamases group including extended spectrum *β-*lactamases (ESBL) was not found in any of the *Aeromonas* strains. Other than *β-*lactamases, the sulfonamide resistance gene, *sul1* and the aminoglycoside resistant gene, *aadA1* were detected in the *A. caviae* SU4-2, and the quinolone resistance gene, *qnrS2* was detected in the *A. rivipollensis* A539. In addition, two genes encoding a major facilitator superfamily (MFS) efflux pump protein, *tet(E)* and *qacEΔ1,* were found in the *A. caviae* SU4-2 while only *tet(E)* was detected in the *A. hydrophila* A537.

**Table 1:** Genotypic and phenotypic antimicrobial resistance (AMR) profile of 19 *Aeromonas* strains isolated from RTE seafood and a salmon processing environment (SPE).

Identified species	Source of isolation	Isolate ID	Genotypic AMR profile					Phenotypic AMR profile	
			β-lactamases			Efflux pump proteins	Other genes	Resistant	Intermediate
			Class B	Class C	Class D				
*Aeromonas caviae*	Retail sushi	SU4-2	–	*bla_MOX-15_*	*bla_OXA-780_*	*tet(E), qacEΔ1*	*sul1, aadA1*	AMP	STX
*Aeromonas dhakensis*	Retail sushi	A533	*cphA2*	*bla_AQU,_*	*bla_OXA-12 (950)_*	–	–	AMP, OA, IPM	–
*Aeromonas hydrophila*	Retail sushi	A537	*cphA2*	*cepS*	*bla_OXA-12 (951)_*	*tet(E)*	–	AMP, OA, EM	–
*Aeromonas rivipollensis*	Retail sushi	A539	–	–	*bla_OXA-427_*	–	*qnrS2*	AMP, OA	–
	Retail sushi	SU3	–	–	*bla_OXA-427_*	–	–	AMP, CTX, OA, EM, FEC	CRO
	Scallop	SC42	–	–	*bla_OXA-427_*	–	–	AMP, EM, FEC	CTX, CRO, OA
	Oyster	OY1	–	–	*bla_OXA-427_*	–	–	AMP, EM, FEC	CRO, OA
*Aeromonas media*	Oyster	OY52	–	*bla_MOX-9_*	*bla_OXA-427_*	–	–	AMP, EM FEC	CTX, CRO, OA
*Aeromonas bestiarum*	Retail sushi	SU6-2	*cphA1*	–	*bla_OXA-956_*	–	–	AMP	IPM
	Salmon loin	SL22	*cphA1*	–	*bla_OXA-956_*	–	–	AMP, IPM, EM	–
	Retail sushi	SU58-3	*cphA1*	–	*bla_OXA-956_*	–	–	AMP	–
*Aeromonas piscicola*	Inlet water (SPE)	LJP308	*cphA1*	–	*bla_OXA-956_*	–	–	AMP, EM	–
*Aeromonas salmonicida*	Retail sushi	SU2	*cphA1*	–	*bla_OXA-956_*	–	–	AMP	IPM
	Salmon loin	SL19	*cphA1, cephA3*	–	*bla_OXA-956_*	–	–	AMP, IPM, EM	–
	Salmon loin	SL21	*cphA5*	–	*bla_OXA-956_*	–	–	AMP, IPM, EM	–
	Scallop	SC45	*cphA5*	–	*bla_OXA-956_*	–	–	AMP, IPM, EM	–
	Oyster	OY56	*cphA1*	–	*bla_OXA-956_*	–	–	AMP, EM, FEC	OA, IPM
	Oyster	OY59	*cphA5*	–	*bla_OXA-956_*	–	–	AMP, IPM, MEM, EM, FEC	OA
	Gutting machine (SPE)	LJP441	*cphA1*	–	*bla_OXA-956_*	–	–	AMP	IPM

AMP: ampicillin, CTX: cefotaxime, CRO: ceftriaxone, OA: oxolinic acid, IMP: imipenem, MEM: meropenem, EM: erythromycin, FEC: florfenicol, and STX: trimethoprim/sulfamethoxazole.

### 3.4. MGEs

**Figure 4:**
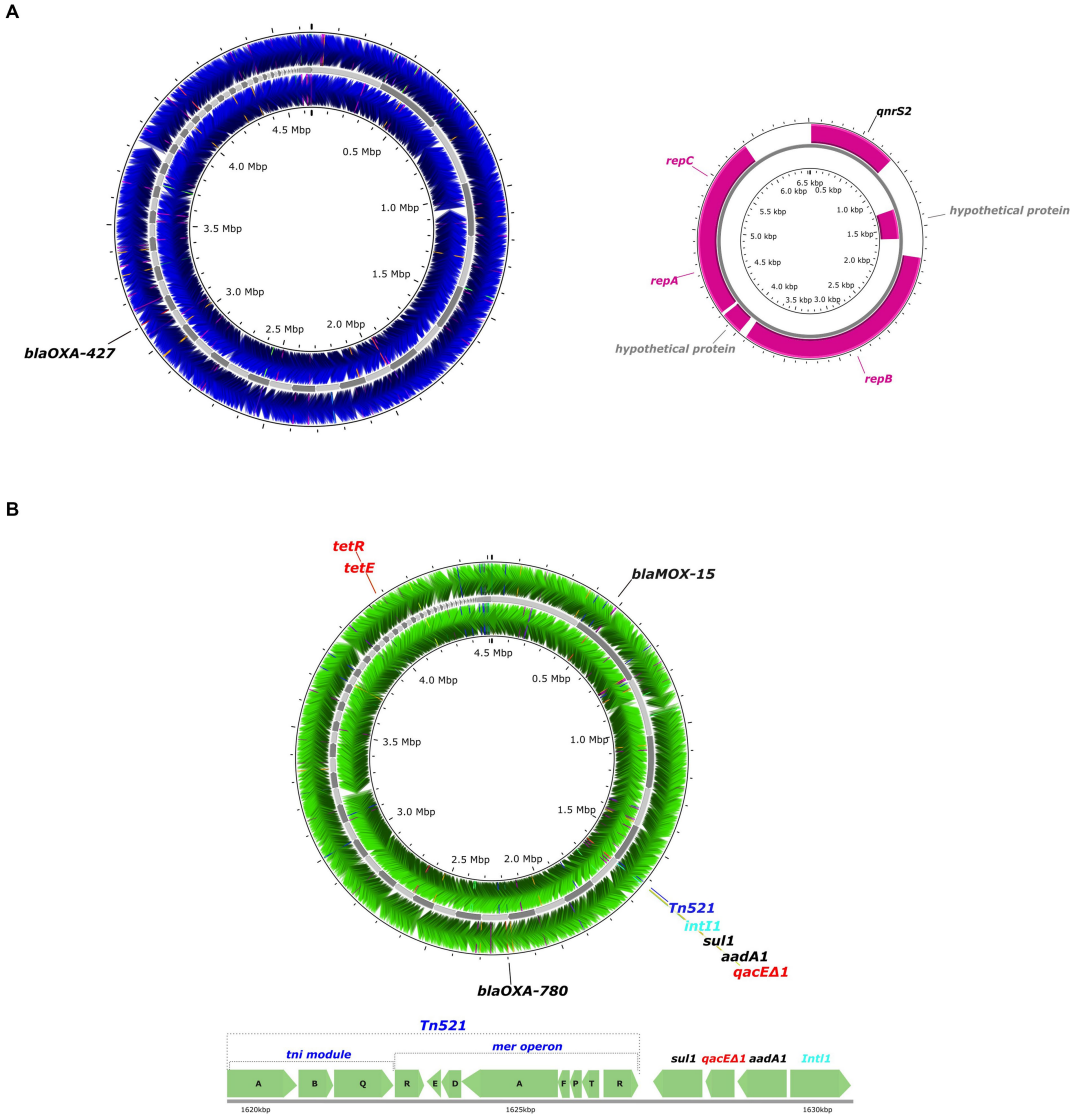
Circular maps of whole genome sequences of the *Aeromonas* A539 and SU4-2 strains isolated from retail sushi products. **(A)** Circular maps of the whole genome sequence of *A. rivipollensis* A539 strain (blue) showing the loci for the resistance gene (black) and its plasmid showing the loci for the resistance genes (black) and replication or mobilization genes (pink). **(B)** A circular map of the whole genome sequence of *Aeromonas caviae* SU4-2 strain (green) showing the loci for the resistance genes (black), a transposon (dark blue), an integron (light blue), and efflux pump proteins (red).

The presence of MGEs including plasmids, transposons, and insertion sequence (IS) elements was examined in the 19 *Aeromonas* strains. *A. rivipollensis* A539 was the only strain that contained a plasmid, which belonged to the IncQ1 group. A circular map of the plasmid (6,535 bp) shows the presence of two replication proteins *repA* and *repC*, the mobilization protein *mobA*, as well as the quinolone resistance gene, *qnrS2* (Figure 4A).

All *Aeromonas* strains except for *A. piscicola* LJP308 carried at least one transposase as a part of their IS elements, which belongs to different IS families such as IS1595, IS3, IS481, IS4, IS1634, IS5, IS21, IS30, IS66, IS110, IS200/IS605, IS256, IS630, and ISAs1 (Supplementary Table S4). Among the 19 strains, the *A. caviae* strain SU4-2 carried the highest number (21) of IS elements, and they were widely distributed in the chromosome. In addition, composite transposons (*cn*) together with their respective IS elements were identified in 12 strains including *A. hydrophila* A537, *A. salmonicida* SL19, OY56, and all strains of *A. bestiarum* and *A. rivipollensis*. In contrast, a non-composite transposon (*Tn*) was only detected in the *A. caviae* strain SU4-2. A circular map of the *A. caviae* SU4-2 genome shows the presence of the transposon *Tn512*, containing the *mer* operon encoding mercury-resistance, as well as a class I integron (*IntI1*; Figure 4B). Two AMR genes *sul1* and *aadA1,* together with an efflux pump gene, *qacEΔ1*, were located between Tn*512* and *IntI.* In addition, a transcription repressor, *tet(R)*, was located next to a tetracycline efflux MFS efflux pump, *tet(E)*.

## 4. Discussion

In this study, 79 *Aeromonas* isolates from RTE seafood and a SPE were subjected to species identification using *gyrB* gene sequencing, and these isolates were tested for susceptibility against 15 antimicrobial agents. Based on the *gyrB* gene sequence identity, 22 isolates representing eight different *Aeromonas* species were selected for WGS considering their source of isolation and phenotypic resistance patterns. A MLPA was performed based on the concatenated sequences of six housekeeping genes in the whole genome sequences of 22 isolates for taxonomy classification. The whole genomes of 19 *Aeromonas* strains were further examined to predict the presence of virulence genes, AMR genes as well as MGEs using different databases.

Identification of *Aeromonas* strains at the species level is still controversial and challenging due to the genetic heterogenicity in the genus *Aeromonas* (Janda and Abbott, 2010; Fernández-Bravo and Figueras, 2020). Molecular identification methods using 16S ribosomal RNA gene or housekeeping genes such as *gyrB* or *rpoD*, have been widely used for defining species and assessing the phylogenetic relationships within genus *Aeromonas* (Yáñez et al., 2003; Soler et al., 2004). Nevertheless, previous studies have shown the limitation of using a single gene sequence to infer the phylogeny of closely related *Aeromonas* species, due to the different phylogenetic resolution of protein-coding genes or possible horizontal gene transfer or recombination (Navarro and Martínez-Murcia, 2018). Currently, a MLPA of concatenated sequences of five or more housekeeping genes has been recommended for the correct identification of *Aeromonas* species (Martínez-Murcia et al., 2011; Navarro and Martínez-Murcia, 2018). In recent years, WGS technology has become more common, and the number of available *Aeromonas* genome sequences in the NCBI database is constantly increasing (Fernández-Bravo and Figueras, 2020). ANI is considered as one of the most robust methods to compare genomic relatedness among strains, with organisms belonging to the same species showing ≥ 95–96% ANI value (Richter and Rosselló-Móra, 2009). Currently, an ANI value of 96% is the recommended cutoff to define species boundaries of *Aeromonas* (Colston et al., 2014). ANI analysis has been used for the taxonomic verification of *Aeromonas* species (Figueras et al., 2014; Beaz-Hidalgo et al., 2015).

In our study, the phylogeny of the 22 isolates were analyzed by the MLPA based on the concatenated sequences of six housekeeping genes. The constructed tree by MLPA showed the robust topology with high supporting values by both NJ and ML methods. The phylogenetic clustering of the 22 isolates to eight different *Aeromonas* species could be verified by the ANI value ≥ 96%. We also constructed the six different phylogenetic trees based on each of the housekeeping gene sequences that extracted from the whole genome sequences. The species identity of 22 isolates based on each of the six gene markers and the concatenated sequences of the six gene markers was identical. One exceptional clustering of SU58-3 with *A. piscicola* was found based on the *dnaX* gene sequence. Nevertheless, we can conclude that the species identity of SU58-3 can be confirmed to be *A. bestiarum* by the ANI value of 97% between SU58-3 and *A. bestiarum*, while the ANI value was less than 95% between SU58-3 and *A. piscicola*. Additionally, the clustering of the *dnaX* gene sequence of the SU 58–3 with *A. piscicola* unlike other gene sequences suggested that using single gene sequence such as *dnaX* gene might not be specific enough to distinguish the closely related species such as *A. piscicola* and *A. bestiarum*.

Mesophilic *Aeromonas* strains are considered emerging pathogens in humans, causing gastrointestinal and extraintestinal infections with various clinical manifestations (Tomás, 2012; Fernández-Bravo and Figueras, 2020). *Aeromonas* pathogenicity is considered a multifactorial process, and the presence of several virulence factors can enable *Aeromonas* to adhere, invade, and destroy the host cells (Tomás, 2012; Fernández-Bravo and Figueras, 2020). Genes related to adherence, motility, immune evasion, secretion, and toxin production were the major virulence factors of *Aeromonas* strains examined in this study.

*Aeromonas* have two independent flagella systems for motility: polar flagella, required for the motility in a liquid environment (swimming) and lateral flagella, which is needed for the movement across a solid surface (swarming) (Kirov et al., 2002). While both flagella systems are involved in the early colonization of human intestinal cells as well as biofilm formation, not all mesophilic *Aeromonas* can produce a lateral flagella system (Kirov et al., 2004). In our study, 47% of 19 *Aeromonas* strains had the genes encoding the lateral flagella including *laf* genes, which are the major genes for lateral flagella biosynthesis (Canals et al., 2006a). However, the absence of the lateral flagella system in the reference strain *A. hydrophila* ATCC 7966 might imply that they might be not absolutely required for the virulence mechanism (Seshadri et al., 2006). Moreover, all 19 strains carried over 50 genes expressing polar flagella, while two major flagellin genes *flaA* and *flaB* were detected only in 15 strains. A previous study by Canals et al. (2006b) showed that the double mutant *flaA* and *flaB* caused the loss of polar flagella and reduced adherence and biofilm formation, implying that only those strains containing these two genes are likely to have the polar flagella with optimal functions.

Among type IV pili detected in mesophilic *Aeromonas* spp., Msh bundle-forming pili are known to be the major adherence system responsible for cell adherence, colonization, and biofilm formation (Kirov et al., 1999; Hadi et al., 2012). A complete gene cluster of Msh pili (*mshA-Q*) observed in ten strains in our study, have been characterized from *A. veronii bv. sobria* and these genes are required for the optimal function of cell adherence and biofilm formation (Hadi et al., 2012). In particular, *mshQ* is known to play an important role in the Msh pili biosynthesis of *A. hydrophila* (Qin et al., 2014). In the genome of *A. hydrophila* ATCC7966, some of the genes *mshA, mshk, mshP* were absent, implying that not all the 17 genes might be required for the adherence function of other species. In addition, genes encoding Tap pili were detected in all strains, including a TapD protein (t*apABCD*), which is known to be associated with the assembly and functionality of Msh pili as well as extracellular secretion of aerolysin and proteases, contributing to the T2SS (Pepe et al., 1996; Hadi et al., 2012). Moreover, the distribution of the genes encoding Flp pili was species-related, since those genes were only detected in the *A. piscicola*, *A. bestiarum*, and *A. salmonicida* strains. While very little is known about the specific role of Flp pili in mesophilic *Aeromonas*, the Flp pili of psychrophilic *A. salmonicida* strains have previously been identified and found not essential for their virulence (Boyd et al., 2008).

Bacterial cell surface polysaccharides including capsules, LPS O-antigens, and S-layers play important roles in immune evasion of many pathogens including *Aeromonas* (Rasmussen-Ivey et al., 2016). In this study, the genes encoding capsules and O-antigens were detected among our *Aeromonas* strains. The capsule covers the outer layers of the bacterial cell wall and is an important virulence factor of *Aeromonas* that helps prevent phagocytosis by host cells and acts as a barrier to toxin substances (Merino and Tomás, 2015). The O-antigen is the most surface-exposed LPS, acting as a colonization factor, and previous studies have shown that *Aeromonas* strains lacking O-antigen were unable to colonize hosts and have reduced expression of T3SS components (Merino et al., 1996; Vilches et al., 2004). Among 19 strains, most of had the gene encoding capsule as a virulence factor, whereas 11 strains representing each of the species except for *A. caviae* having both capsule and O-antigen, are likely involved in the virulence mechanisms of colonizing and invading host cells. However, none of the genes encoding capsule or O-antigen were detected in two reference genomes based on the VFDB. Considering that LPS O-antigen structure of the *A. salmonicida* strains A449 was previously characterized by the previous study (Wang et al., 2005), we cannot rule out the presence of those genes in both reference genomes. It might be that current database of VFDB is not fully updated with the genes associated with capsules or O-antigens of *Aeromonas*.

Of six secretion systems identified in Gram-negative bacteria, only T2SS, T3SS and T6SS were detected in the 19 strains. T2SS (detected in all strains) is an essential pathway for the pathogenesis of *Aeromonas*, since it is involved in the extracellular secretion of virulence factors such as DNase, protease, hemolysin and aerolysin (Sandkvist, 2001; Lowry et al., 2014). Both T3SS and T6SS are considered virulence markers of *Aeromonas* (Tomás, 2012). T3SS is a needle-like structure, injecting effectors directly into host cells, different T3SS effectors such as AexT, AexU, AopP, AopO and AopH have previously been identified in virulent *A. hydrophila* and *A. salmoncidia* strains (Burr et al., 2003; Sha et al., 2007). On the other hand, other studies have shown that both environmental and epidemic *A. hydrophila* strains lacked the genes encoding T3SS, implying that alternate secretion system is more critical for the virulence of *A. hydrophila* and probably other species as well (Seshadri et al., 2006; Pang et al., 2015). Like T3SS, T6SS could also inject their effectors into the host cells, and four effectors including Hcp (hemolysin coregulated protein), VgrG (valine-glycine repeat protein G), vasH (sigma factor 54 activator) and vasK are typical characteristics of the T6SS (Suarez et al., 2008, 2010). A previous study has showed that deletion of two genes *hcp1* and *vgG1* in virulent *A. hydrophila* strains significantly reduced their virulence (Tekedar et al., 2019). Besides, the T6SS has also been characterized from non-pathogenic *Aeromonas* strains (Pang et al., 2015; Rasmussen-Ivey et al., 2016). In our study, the T6SS including two genes (*hcp1* and *vgG1*) was highly prevalent among the strains, implying their potential roles in pathogenicity and biofilm formation, while most of the strain lacked the genes encoding T3SS.

Two main types of enterotoxins are present in *Aeromonas* spp.: cytotoxic and cytotonic (Tomás, 2012). Cytotoxic enterotoxins include Act and aerolysin, known as the main virulence factors of *A. hydrophila*, and responsible for hemolytic, cytotoxic and enterotoxic activity (Chopra et al., 1993; Chopra and Houston, 1999). Two types of cytotonic enterotoxin include heat-liable Alt, and heat-stable Ast (Chopra et al., 1994). Hemolysins are cytotoxic and pore-forming toxins produced by pathogenic bacteria, and two hemolysin genes (*hlyA* and *aerA*) have been detected from all virulent *A. hydrophila* strains from the previous studies (Wong et al., 1998; Heuzenroeder et al., 1999). In addition, a previous study by Wang et al. (2003) showed that the combination of *aerA* and *ahh1* genes seemed to be the most cytotoxic genotype of hemolysins, identified from all virulent *A. hydrophila*. In our study, three hemolysin genes encoding hemolysinA (*hlyA*), thermostable hemolysins (*th*) and hemolysinIII (*hlyIII*) were detected in all strains. Both *aerA* and *ahh1* encoding aerolysin and extracellular hemolysin were detected in the *A. dhakensis*, *A. hydrophila*, *A. bestiarum*, *A. piscicola* and *A. salmonicida* strains, implying their potential for cytotoxic effects. Moreover, a RTX operon consisting of six genes (*rtxACHBDE*) were detected in some of the *A. hydrophila*, *A. dhakensis*, *A. bestiarum* and *A. salmonicida* strains, while only *rtxA* (exotoxin) was detected in the *A. rivipollensis* strains. A previous study by Suarez et al. (2012) showed that *rtxA* plays an important role in host cell rounding and apoptosis.

Our data showed the presence of multiple AMR genes in the *Aeromonas* strains regardless of the source of isolation. A large proportion of AMR genes detected in our *Aeromonas* strains belonged to the Ambler class B, C, and D *β*-lactamases, and species-specific distribution of *β-*lactamases genes was observed in accordance with previous observations (Fosse et al., 2003; Chen et al., 2012; Dubey et al., 2022a). *Aeromonas* spp. could produce *β*-lactamases which confer resistance to a broad spectrum of *β*-lactams antibiotic by hydrolyzing the four-membered *β-*lactam ring of antibiotics, and Ambler class B, C, and D *β*-lactamases are known as three major classes of chromosomally mediated β-lactamases detected in *Aeromonas* (Janda and Abbott, 2010).

Among the Ambler class B *β-*lactamases, the most prevalent gene among the 19 *Aeromonas* genomes was *cphA*. Previous research has reported that *cphA* gene is considered intrinsic among environmental *Aeromonas* spp., showing carbapenems-hydrolyzing activity (Balsalobre et al., 2009). In our study, cphA *β-*lactamases such as *cphA1*, *cphA2*, or *cphA5* were detected in the chromosome of several strains, which showed phenotypic resistance or reduced susceptibility to imipenem or meropenem (carbapenems). The presence of those genes in the *Aeromonas* strains is likely to confer their phenotypic resistance to carbapenems. In addition, we observed the species-specific distribution of *cphA* genes, in accordance with previous studies (Rossolini et al., 1995; Chen et al., 2012). The class B *β-*lactamase genes, particularly *cphA* seem to be prevalent among *Aeromonas* spp., as these genes have frequently been observed in both environmental and clinical isolates of *Aeromonas* (Rossolini et al., 1995; Balsalobre et al., 2009; Wu et al., 2012).

Of the 19 strains, only four strains contained the genes belonging to the Ambler class C *β*-lactamases, associated with the resistance mechanisms of many β-lactam antibiotics, including narrow spectrum cephalosporins, third generation cephalosporins, but less active on fourth generation cephalosporins (Bush et al., 1995). Class C *β*-lactamases have been identified in both chromosomes and plasmids of *Aeromonas* spp. originating from various sources (Piotrowska et al., 2017; Ebmeyer et al., 2019; Piccirilli et al., 2022). The class C *β*-lactamases genes detected in our study were *bla_MOX-9_* (in *A. media*), *bla_MOX-15_* (in *A. caviae*), *bla_AQU_* (in *A. dhakensis*), and *cepS* (in *A. hydrophila*). Among these, *bla_MOX_* variants from *bla_MOX-3_* to *bla_MOX-12_* have previously been found in both environmental and clinical isolates of *Aeromonas* spp. (Ye et al., 2010; Piotrowska et al., 2017). In addition, *bla_MOX-9_* was previously found in the transposon of *A. media* species and considered as a mobile antibiotic resistance gene (Ebmeyer et al., 2019; Piccirilli et al., 2022), whereas this gene detected in our study was encoded in the chromosome of the *A. media* strain. Moreover, the presence of *bla_AQU_* was observed in clinical isolates of *A. dhakensi*s showing cefotaxime resistance (Wu et al., 2013), and *cepS* together with *bla_OXA-12_* and *cphA7* genes were detected in clinical strains of *A. hydrophila* strains showing carbapenem resistance (Hilt et al., 2020). Both *bla_AQU_* and *cepS* have also been detected in environmental isolates of *Aeromonas* spp. (Wang et al., 2021; Dubey et al., 2022a).

Among the Ambler class D *β*-lactamases, oxacillin-hydrolyzing type β-lactamases (OXAs) was observed in all 19 strains. OXAs can confer resistance not only to penicillin, but also to cephalosporins and carbapenems (Evans and Amyes, 2014). OXAs have been identified among several Gram-negative bacteria, including *Aeromonas* spp. (Poirel et al., 2010). The first OXA-like gene identified in the chromosome of *Aeromonas* spp. was designated as *bla_OXA-12_*, and *bla_OXA-12_* associated genes including new variants such as *bla_OXA-427_*, *bla_OXA-780_*, *bla_OXA-830_* and *bla_OXA-956_* are considered innate in *Aeromonas* spp. (Rasmussen et al., 1994; Chen et al., 2019). Accordingly, four different *bla_OXA_* genes (*bla_OXA-12_*, *bla_OXA-427_*, *bla_OXA-780_*, *bla_OXA-956_*) detected in our study were chromosomally encoded in the *Aeromonas* genomes, and a species-related distribution was observed. Previous studies have also reported the presence of *bla_OXA_* genes in the *Aeromonas* spp. isolated from environmental (Moura et al., 2012; Piotrowska et al., 2017) and clinical samples (Hilt et al., 2020; Tang et al., 2020).

Other than *β*-lactamases, the quinolone resistance gene *qnrS2* was present in the *A. rivipollensis* strain A539 showing phenotypic resistance to quinolones. Furthermore, the sulfonamide resistant gene *sul1* was detected in the *A. caviae* strain SU4-2, that phenotypically showed reduced susceptibility to trimethoprim/sulfamethoxazole. On the other hand, some discrepancies between genotypic and phenotypic resistance were observed. For instance, phenotypic resistance to erythromycin or florfenicol could not be predicted based on the AMR gene profiles, and no correlation was found between the aminoglycoside resistance gene, *aadA1* and the phenotypic resistance of the *A. caviae* strain SU4-2. Both AMRFinder and ResFinder databases have been constructed with high accuracy in predicting genotype–phenotype concordance for some foodborne pathogens; however, the outcomes may depend on the bacterial species, the type of antibiotics and the associated mechanism of resistance (Feldgarden et al., 2019; Bortolaia et al., 2020). In addition, incorrect phenotypic data might be the reason for discrepancies, since repeating phenotypic testing could have resolved most discrepancies between phenotypes and predicted genotypes using ResFinder (Zankari et al., 2013). Moreover, multidrug efflux pump proteins were detected from the two strains *A. caviae* SU4-2 and *A. hydrophila* A537. The MFS efflux pump gene, *qacEΔ1* linked to sulfonamide (*sul1*) and aminoglycoside (*aadA1*) resistance genes was observed in the *A. caviae* strain SU4-2, while another efflux pump gene, *tet(E)* linked to the *tet(R)* was found in both *A. caviae* SU4-2 and *A. hydrophila* A537 strains. Similarly, the *qacEΔ1* together with other resistance genes (*sul1*, *aadA2*) were also detected in *A. caviae* and *A. hydrophila* strains isolated from fish (Dubey et al., 2022a). In addition, the *tet(E)* has previously been identified in *Aeromonas* (Dubey et al., 2022b; Erickson et al., 2023).

A pool of genes located in MGEs such as plasmids, transposons, and integrons are considered flexible and transferrable, and may be associated with virulence factors, toxic compounds as well as antibiotic resistance (Piotrowska and Popowska, 2015). Several studies have reported the presence of MGEs in *Aeromonas* strains isolated from aquatic environments and fish, and their association with resistance or virulence determinants (McIntosh et al., 2008; del Castillo et al., 2013; Dubey et al., 2022a; Song et al., 2023). In our study, an IncQ plasmid carrying *qnrS2* was detected in the *A. rivipollensis* strain A539 isolated from a retail sushi product. Other studies have suggested the strong relationship between the IncQ plasmid and *qnrS2*, where they have observed the IncQ plasmids harboring *qnrS2* in *Aeromonas* strains isolated from fish and water (Cattoir et al., 2008; Majumdar et al., 2011; Han et al., 2012). Moreover, we found the co-localization of a transposon Tn521 containing a mercury operon and a class I integron (*Int11*) with two AMR genes (*sul1, aadA1*) and an efflux pump protein (*qacEΔ1*) in the *A. caviae* strain SU4-2, implying co-resistance of mercury and antibiotic resistance. Co-resistance occurs when resistance determinants to heavy metals and antibiotics are harbored together in the same MGEs, and this co-localization could result in co-selection mechanisms of other genes in the same elements (Chapman, 2003; Baker-Austin et al., 2006). Some studies have reported the IncA/C plasmids of *Aeromonas* spp. carrying the mercury operon and AMR genes in the class I integron (McIntosh et al., 2008; del Castillo et al., 2013;). Besides, several transposons and IS elements detected in *Aeromonas* spp. have strongly been associated with *β*-lactamases in previous studies (Girlich et al., 2011; Picão et al., 2013). However, we found no strong association between other MGEs identified in our *Aeromonas* strains and AMR genes.

## 5. Conclusion

In the present study, 22 *Aeromonas* strains isolated from RTE seafood and a SPE were classified into eight different *Aeromonas* species based on the MLPA and ANI analysis. Most strains contained several genes encoding major virulence factors related to adherence, motility, immune evasion, secretion systems, and toxins, and in particular, we observed the high incidence of enterotoxins, T6SS and its major effectors. Multiple AMR genes encoding class B, C and D *β*-lactamases were found in all *Aeromonas* strains and their distribution was species-related. In addition, the presence of other AMR genes located in MGEs such as an IncQ type plasmid in the *A. rivipollensis* strain A539 and a transposon and a class I integron in the *A. caviae* SU4-2 indicates their potential to disseminate AMR genes to other bacteria. Considering that most *Aeromonas* strains were isolated from RTE seafood, our study suggests that *Aeromonas* strains circulating in the food chain could potentially be pathogenic and act as a vector for dissemination of AMR genes to other bacteria residing in the same environments. Thus, we highlight the importance of collecting more knowledge of AMR and mobilome in the genus *Aeromonas* to understand their ability to transfer AMR within the food chain, and the potential risk of AMR caused by *Aeromonas* circulating in the food chain should be carefully monitored.

## Data availability statement

The datasets presented in this study can be found in online repositories. The names of the repository/repositories and accession number(s) can be found in the article/Supplementary material and in the NCBI database under accession number: PRJNA877469.

## Author contributions

H-JL: manuscript preparation, methodology, bioinformatics analysis, editing, and submission. JS: methodology, bioinformatics analysis, and editing. JL: editing and supervision. B-TL, SH, and AJ: conceptualization, editing, and supervision. All authors contributed to the article and approved the submitted version.

## Funding

This work was funded by Norwegian University of Science and Technology (NTNU). H-JL was supported by a PhD grant from NTNU, as part of the OPTiMAT project.

## Conflict of interest

The authors declare that the research was conducted in the absence of any commercial or financial relationships that could be construed as a potential conflict of interest.

## Publisher’s note

All claims expressed in this article are solely those of the authors and do not necessarily represent those of their affiliated organizations, or those of the publisher, the editors and the reviewers. Any product that may be evaluated in this article, or claim that may be made by its manufacturer, is not guaranteed or endorsed by the publisher.

## Footnotes

^1^ https://jgi.doe.gov/data-and-tools/software-tools/bbtools/bb-tools-user-guide/bbduk-guide/
