# Supplementary material for: Whole genome sequence analysis of Aeromonas spp. isolated from ready-to-eat seafood: antimicrobial resistance and virulence factors
Source: Front Microbiol. 2023 Jun 30;14:1175304. doi: 10.3389/fmicb.2023.1175304 (PMC10348363; doi:10.3389/fmicb.2023.1175304)
Supplement: Supplementary file 1 [file Data_Sheet_1.docx]

Supplementary Material

Genomic characterization of Carbapenem-resistant Klebsiella oxytoca complex in China: a multi-center study

**Weimin Wan^1,2†^, Xiaochun Yang^2†^, Hua Yu^3^, Min Wang^1^, Wei Jia^4^, Bin Huang^5^, Fen Qu^6^, Bin Shan^7^, Yi-Wei Tang^8^, Liang Chen^9^, Hong Du^1*^**

*** Correspondence:** Corresponding Author: [hong_du@126.com](mailto:hong_du@126.com) (H. Du)


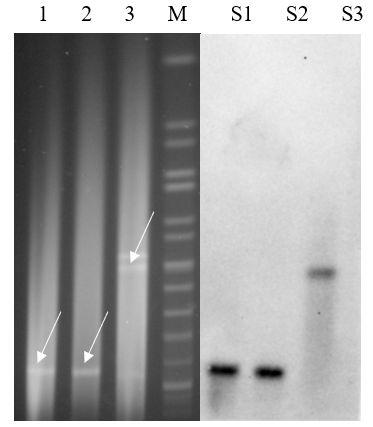


**Supplementary Figure 1: Plasmid S1-PFGE and Southern blot-IMP.** Lanes 1-3 are S1-PFGE; Lanes S1-S3 are Southern blot; M is the marker H9812. Lanes 1-3 are *K. ox*1344, *K. ox*3873, *K. ox*3097, respectively


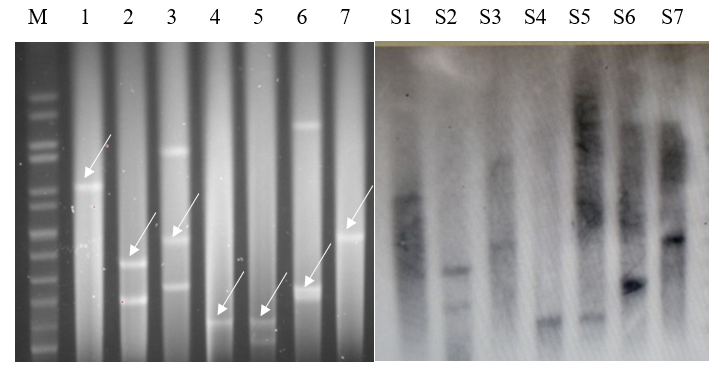


**Supplementary Figure 2: Plasmid S1-PFGE and Southern blot-NDM.** Lanes 1-7 are S1-PFGE; Lanes S1-S7 are Southern blot; M is the marker H9812. Lanes 1-7 are *K. ox*1352, *K. ox*3867, *K. ox*4353, *K. ox*1962, *K. ox*2641, *K. ox*2871, *K. ox*1688 respectively.


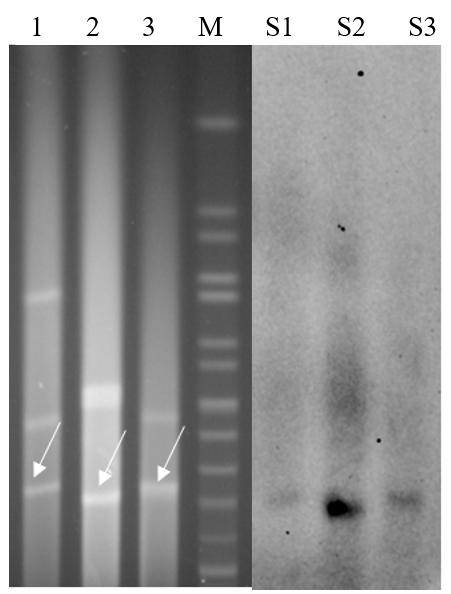


**Supplementary Figure 3: Plasmid S1-PFGE and Southern blot-KPC.** Lanes 1-3 are S1-PFGE; Lanes S1-S3 are Southern blot; M is the marker H9812. Lanes 1-3 are *K. ox*4353, *K. ox*2691, *K. ox*4379 respectively.

**Supplementary Table 1: MICs of CRKO complex strains against carbapenems**

| **Isolates** | **Imipenem** | **Meropenem** |
| --- | --- | --- |
| *K. ox*1962 | 128 | 64 |
| *K. ox*2641 | 128 | 32 |
| *K. ox*2691 | 64 | 16 |
| *K. ox*4379 | 64 | 8 |
| *K. ox*4543 | 32 | 4 |
| *K. ox*6089 | <=0.25 | 4 |
| *K. ox*6177 | 4 | 2 |
| *K. ox*7419 | 64 | 16 |
| *K. ox*2871 | 64 | 64 |
| *K. ox*5230 | 64 | 32 |
| *K. ox*1688 | 64 | 32 |
| *K. ox*1344 | 32 | 8 |
| *K. ox*1352 | 64 | 16 |
| *K. ox*2349 | 32 | 16 |
| *K. ox*2708 | 64 | 32 |
| *K. ox*3097 | 16 | 16 |
| *K. ox*3867 | 128 | 32 |
| *K. ox*3873 | 16 | 8 |
| *K. ox*4353 | 128 | 32 |
| *K. ox*6915 | 16 | 16 |
| *K. ox*7525 | 2 | 8 |
| *K. ox*7566 | 16 | 8 |
| *K. ox*7768 | 32 | 8 |
| *K. ox*8405 | 64 | 16 |
| *K. ox*8859 | 4 | 2 |

**Supplementary Table 2: Primer sequences of hybridization probes.**

| **Target genes** | **Sequence (5’-3’)** | **Amplicon size (bp)** |
| --- | --- | --- |
| *bla*_KPC_ | F*:* ACGGCCTTCATGCGCTCTATC  R*:* AATTGTAATACGACTCACTATAGGGCG  GTCGTGTTTCCCTTTAGCCAA | 178 |
| *bla*_NDM_ | F: CCGACGATTGGCCAGCAAATG  R: AATTGTAATACGACTCACTATAGGGCG  GGATCTGGGCGGTCTGGTCAT | 164 |
| *bla*_IMP_ | F: TCCCCACGTATGCATCTGAA  R: GCAGCCAAACCACTACGTTA | 163 |
